# Notes and News

**Published:** 1897-04-03

**Authors:** 


### NOTES AND NEWS.

(Collected and Communicated.)

The foundation-stone of a new wing at the Horton Infirmary has been laid. The whole expense of the addition is being defrayed by Mr. W. Thewburn, at the cost of £1,000. The ground floor will contain two wards, a nurses' room, bath-room, kitchen, and offices. On the first floor there will be an isolation ward, nurses' room, and offices. The wing will be connected to the main building by a passage. The whole accommodation is to be devoted to children.

The following letter from the Local Government Board, published in *London* last week, will be of interest to many dwellers in country districts, showing as it does that the workhouse infirmaries may properly be utilised as accident hospitals, where such do not exist in the immediate neighbourhood:—

"Whitehall.

"Sir,—I am directed by the Local Government Board to acknowledge the receipt of your letter of the 17th ultimo, and in reply to state that they have not issued any order to the effect stated therein.

"I am, however, to add that a master of a workhouse or a medical superintendent of an infirmary may admit persons in case of sudden and urgent necessity (such as accidents) without any order from a relieving officer.—Yours, &c.,

"W. E. Knollys."

The medical atmosphere of Accrington appears to be of a disturbing description. Some remarkable transactions have centred round the Medical Dispensary. It is not easy to get at the true inwardness of the affairs which culminated in an indignation meeting of members and inhabitants of Accrington recently. During the course of the meeting it transpired that the dispensary was suddenly abolished, the furniture sold at a nominal sum, the drugs (valued differently by various witnesses) totally "disappeared," and the members were reported as "handed over" to the Accrington Medical Society and the doctor discharged. At the meeting all was confusion and contradiction, but eventually a business-like result was obtained, and a resolution was passed censuring the general proceedings of the committee and the disregard of rules, and advocating that the papers and documents of the dispensary be laid before the Registrar of Friendly Societies with a view to complete investigation. Finally the committee of the dispensary was reconstituted, thus commencing the re-establishment of the institution.

That beneficent work, the Factory Girls' Holiday Fund, continues to do good work and to prosper. The girls and the women themselves contribute to the best of their ability, and thus the pleasure of the holiday is enhanced. Arrangements are made by kind friends in different parts of the country for the lodging of the visitors, and much help can be rendered by those who will undertake to suggest places where the girls can be received and where they can be supervised during their stay. The fund was established in 1888, when 39 girls were benefited. During 1896, 1,050 girls and women enjoyed a week or two of holiday through its means. The honorary secretary is Miss Canney, of St. Peter's Rectory, Saffron Hill, E.C.

Last week we drew attention to the alarming frequency of deaths from anæsthetics in England, and especially from chloroform. At the time the article was written 20 deaths from chloroform had taken place this year, but in the few days that elapsed two more deaths had occurred, making it necessary to change our figure to 22. Since then, however, the following four more deaths have occurred: One in Birmingham, from chloroform; one at Leicester, also from chloroform; one at Windsor, under gas and ether; and one at Liverpool, under a mixture of alcohol, chloroform, and ether. This makes 26 cases of death in three months which have come to our knowledge; how many more may have occurred without being reported we cannot say. It is a very serious matter.

There are two fatalities which appear constantly in the columns of our newspapers, namely, deaths caused by the overturning or explosion of paraffin lamps, and those resulting from the consumption of tinned food. Public opinion is gradually coming to the conviction that something should be done to reduce the dangers arising from the former, but shows itself very apathetic in respect to the latter. Tinned food, especially salmon, is largely consumed by the poor, and it is therefore as essential that this class of food should be subject to the same scrutiny as meat and other commodities liable to rapid decomposition. But this is not so, and there can be no question that a large amount of sickness is due to unwholesome tinned food. In a recent issue of the *Globe* a most appalling account of the salmon tinning industry on the Fraser river is given. The article is signed, and the writer expresses himself willing to substantiate all his charges. He points out the impossibility of the public being able to distinguish the different kinds, and therefore shows that the erring companies endanger the whole trade. Certainly it is impossible to consume tinned salmon with any satisfaction after reading the article in question. Putrid fish, foul water used to "cleanse" the fish, and dirty Chinese coolies employed in the preparation of the same, and further the report of employés dying of typhoid fever present a haunting picture which will cause many to avoid tinned salmon, at any rate until official inspection can return a clean bill of health once more. Mr. Wood, who writes on the subject, states that with a little less greed on the part of the exporters, the production of properly prepared and wholesome tinned salmon is quite possible.

